# Cardiac myxoma in pregnancy: a comprehensive review

**DOI:** 10.5935/1678-9741.20150012

**Published:** 2015

**Authors:** Shi-Min Yuan

**Affiliations:** 1 The First Hospital of Putian, Teaching Hospital, Fujian Medical University, Putian, Fujian Province, People’s Republic of China.

**Keywords:** Cesarean Section, Myxoma, Fetal Mortality

## Abstract

**Objective:**

Cardiac myxoma in pregnancy is rare and the clinical characteristics of this entity have been insufficiently elucidated. This article aims to describe the treatment options and the risk factors responsible for the maternal and feto-neonatal prognoses.

**Methods:**

A comprehensive search of the literature of cardiac myxoma in pregnancy was conducted and 44 articles with 51 patients were included in the present review.

**Results:**

Transthoracic echocardiography was the most common diagnostic tool for the diagnosis of cardiac myxoma during pregnancy. Cardiac myxoma resection was performed in 95.9% (47/49); while no surgical resection was performed in 4.1% (2/49) patients (*P*=0.000). More patients had an isolated cardiac myxoma resection in comparison to those with a concurrent or staged additional cardiac operation [87.2% (41/47) *vs.* 12.8% (6/47), *P*=0.000]. A voluntary termination of the pregnancy was done in 7 (13.7%) cases. In the remaining 31 (60.8%) pregnant patients, cesarean section was the most common delivery mode representing 61.3% and vaginal delivery was more common accounting for 19.4%. Cardiac surgery was performed in the first, second and third trimester in 5 (13.9%), 14 (38.9%) and 17 (47.2%) patients, respectively. No patients died. In the delivery group, 20 (76.9%) neonates were event-free survivals, 4 (15.4%) were complicated and 2 (7.7%) died. Neonatal prognoses did not differ between the delivery modes, treatment options, timing of cardiac surgery and sequence of cardiac myxoma resection in relation to delivery.

**Conclusion:**

The diagnosis of cardiac myxoma in pregnancy is important. Surgical treatment of cardiac myxoma in the pregnant patients has brought about favorable maternal and feto-neonatal outcomes in the delivery group, which might be attributable to the shorter operation duration and non-emergency nature of the surgical intervention. Proper timing of cardiac surgery and improved cardiopulmonary bypass conditions may result in even better maternal and feto-neonatal survivals.

## Abstract (pt)

**Objetivo:**

Mixoma cardíaco durante a gravidez é raro e as características clínicas dessa entidade não foram suficientemente esclarecidas. Este artigo tem como objetivo descrever as opções de tratamento e os fatores de risco responsáveis pelo prognóstico materno e fetal-neonatal.

**Métodos:**

Foi realizada uma pesquisa abrangente na literatura sobre mixoma cardíaco durante a gravidez e 44 artigos com 51 pacientes foram incluídos na presente revisão.

**Resultados:**

Ecocardiografia transtorácica foi a ferramenta de diagnóstico mais comum para o diagnóstico de mixoma cardíaco durante a gravidez. Ressecção do mixoma cardíaco foi realizada em 95,9% (47/49); enquanto não foi realizada ressecção cirúrgica em 4,1% (2/49) dos pacientes (*P*=0,000). Mais pacientes tiveram ressecção isolada do mixoma cardíaco em comparação com aqueles com operação cardíaca concomitante ou adicional [87,2% (41/47) *vs.* 12,8% (6/47), *P*=0,000]. A interrupção voluntária da gravidez foi feita em 7 (13,7%) casos. Nas restantes 31 (60,8%) pacientes grávidas, a cesariana foi o modo de parto mais comum, representando 61,3% e parto vaginal contabilizou 19,4%. A cirurgia cardíaca foi realizada no primeiro, segundo e terceiro trimestre em 5 (13,9%), 14 (38,9%) e 17 (47,2%) pacientes, respectivamente. Nenhuma paciente morreu. No grupo de parto, 20 (76,9%) recém-nascidos sobreviveram livres de eventos, 4 (15,4%) tiveram complicações e 2 (7,7%) morreram. Os prognósticos neonatais não diferiram entre os modos de parto, opções de tratamento, tempo de cirurgia cardíaca e sequência de ressecção mixoma cardíaco em relação ao parto.

**Conclusão:**

O diagnóstico de mixoma cardíaco durante a gravidez é importante. Tratamento cirúrgico de mixoma cardíaco em pacientes grávidas trouxe resultados favoráveis para as mães e os neonatos no grupo de parto, o que pode ser atribuído à duração mais curta da operação e à natureza não emergencial da intervenção cirúrgica. O momento adequado da cirurgia cardíaca e melhoria das condições de circulação extracorpórea podem resultar em sobrevivência materna e do feto-neonato ainda melhor.

**Table:** 

**Abbreviations, acronyms & symbols**	
CMCI	Chinese Medical Citation Index
QUOROM	Quality of Reporting of Meta-Analyses

## INTRODUCTION

Cardiovascular disorders during pregnancy have become a more and more attracting issue concerning both mother and child in terms of their prognoses^[1]^. Cardiac surgery during pregnancy remains a tough problem due to the fact that cardiopulmonary bypass jeopardizes fetuses more than mothers^[2]^. The overall feto-neonatal mortality was 18.6% among the pregnant patients with a cardiac operation^[1]^. The fetal deaths were apparently associated with cardiac surgery during early pregnancy as well as the use of cardiopulmonary bypass^[3]^. Cardiac myxoma in pregnancy is one of the cardiovascular disorders that warrant a surgical resection without delay^[1]^. However, the clinical features of cardiac myxomas in the pregnant patients have not been sufficiently elaborated, and the risk factors influencing the maternal and feto-neonatal outcomes remain uncertain. In order to highlight these aspects, a comprehensive literature review of pregnant cardiac myxoma is conducted.

## METHODS

Publications in all languages reporting on cardiac myxoma during pregnancy until November 2014 were retrieved from MEDLINE, Highwire Press, Google and Yahoo! search engines, Chinese Medical Citation Index (CMCI) and LILACs. The search terms "cardiac myxoma" and "pregnancy" were searched. In addition, "left atrial", "right atrial", "left ventricular", "right ventricular", "mitral valve", "tricuspid valve" and "aortic valve" were also used in the search strategy to find articles containing cardiac myxomas.

Primary exclusion criteria included articles describing cardiac myxoma diagnosed postpartum, other types of cardiac tumors, undetermined nature of intracardiac mass, or myxoma of the other organs of the pregnant patients, fetal cardiac myxoma, or cardiac myxoma of animals. Papers with no complete data of the pregnant patients were excluded for the statistical analyses. Table 1 shows the results of literature retrieval. Data were carefully extracted for details of the patient population, demographics, diagnosis, clinical features of cardiac myxomas, associated disorders/comorbidities, cardiac surgical procedures, delivery modes, timing of cardiac myxoma and delivery, follow-up length and survival, complication and mortality of both mother and baby, etc. This rare condition was only reported in sporadic single case or small series without larger patient population, comparative, or randomized studies. Accordingly, the qualitative analysis of the collective data from the retrieved articles constituted a systematic review, as suggested in the Quality of Reporting of Meta-Analyses (QUOROM) recommendations^[4]^.

**Table 1:** Literature retrieval.

Database	Total	Irrelevant	Relevant	Inclusive (by excluding duplicate indexes)
PubMed	58	34	24	26
Google	90	34	56	11
Yahoo!	120	94	26	3
Highwire Press	662	659	3	3
Chinese Medical Citation Index (CMCI)	27	23	4	4
LILACs	2	2	0	0

Quantitative data were presented as mean ± standard deviation along with range and median values, and intergroup differences were compared by unpaired t-test. Comparisons of frequencies were made by Fisher's exact test and *P*<0.05 was considered statistically significant.

## RESULTS

### Patient information

A total of 47 articles were collected. By excluding 3 duplicate publications^[5-7]^, 44 articles^[2,8-50]^ involving 51 pregnant patients were taken for statistical analysis. Their ages were 29.5±5.1 (20-40; median, 29) (*n*=39). Their pregnant history was not mentioned in 21 (41.2%) patients, while reported in 30 (58.8%) patients with 8 (26.7%) nulliparous, 8 (26.7%) monoparous and 14 (46.7%) multiparous. The timing of the pregnant patients to be symptomatic was available for 15 (29.4%) patients, with a mean duration of diseased course of cardiac myxoma of 4.0±7.2 months (28 hours-24 months; median, 1 month) (*n*=10) (no quantitative timing was available in 3 patients^[11,19,39]^.

### Clinical features

The symptoms of the cardiac myxoma of the pregnant patients were described in 39 (76.5%) patients. Eight (20.5%) patients were asymptomatic^[12,16,21,35,41,42,45,48]^; while 31 (79.5%) manifested one or two of the Goodwin's triad, namely circulatory, embolic and constitutional symptoms (Table 2). A cardiac murmur or an abnormal heart sound was audible in 18 (35.3%) patients with a systolic murmur [6 (33.3%)]^[12,15,26,34,35,43]^ and a diastolic murmur [4 (30.8%)]^[8,14,33,45]^ being the most common one. Twenty-seven (52.9%) patients had one or more comorbidities or complications of a cardiac myxoma (Table 3).

**Table 2:** Presenting symptoms of 31 pregnant patients.

**Symptom**		***n* (%)**	**References**
**Circulatory**	**15 (48.4)**		
Dyspnea, palpitation		5 (33.3)	^[20,26,28,47,50]^
Chest pain, dyspnea/tachypenia		4 (26.6)	^[10,13,32,34]^
Pulmonary edema		2 (13.3)	^[9,18]^
Dyspnea		1 (6.7)	^[26]^
Palpitation		1 (6.7)	^[29]^
Hemoptysis, dyspnea and cough		1 (6.7)	^[27]^
Dyspnea, pulmonary edema		1 (6.7)	^[40]^
**Circulatory + constitutional**	**7 (22.6)**		
Palpitations, dyspnea, fatigue		2 (28.6)	^[30,39]^
Cough, exhaustion		2 (28.6)	^[19,46]^
Palpitation, fatigue, weight loss		1 (14.3)	^[14]^
Palpitation, dyspnea, night sweat		1 (14.3)	^[33]^
Chest pain, palpitation, cough, fever, chills		1 (14.3)	^[24]^
**Embolic**	**4 (12.9)**		
Headache, memory loss		1 (25)	^[49]^
Transient ischemic attack, weakness		1 (25)	^[38]^
Blurred vision		1 (25)	^[2]^
Hemiparesis, optalmoplegia		1 (25)	^[29]^
**Circulatory + embolic**	**3 (9.7)**		
Dyspnea, orthopnea, dizziness		1 (33.3)	^[36]^
Chest pain, psychomotor restlessness		1 (33.3)	^[11]^
Chest pain, dyspnea, palpitation, syncope, dizziness		1 (33.3)	^[15]^
**Embolic + constitutional**	**1 (3.2)**		
Dizziness, fever		1 (100)	^[8]^
**Circulatory + embolic + constitutional**	**1 (3.2)**		
Dyspnea, dizziness, fatigue		1 (100)	^[43]^

**Table 3:** Associated disorders.

**Associated disorder**	***n* (%)**	**References**
Stroke/embolic events	4 (14.8)	^[2,15,38,49]^
Recurrent cardiac myxoma	3 (11.1)	^[12,35,41]^
Pulmonary edema	3 (11.1)	^[9,18,40]^
Mitral stenosis	2 (7.4)	^[26,28]^
Mitral valve prolapse	2 (7.4)	^[13,26]^
Ventricular tachycardia/premature	2 (7.4)	^[21,42]^
Pulmonary hypertension	1 (3.7)	^[10,47]^
Non-ST-segment elevation myocardial infarction	1 (3.7)	^[24]^
Mitral stenosis, pulmonary hypertension	1 (3.7)	^[23]^
Hodgkin’s disease	1 (3.7)	^[34]^
Diabetic nephropathy, preeclampsia	1 (3.7)	^[11]^
Right adrenalectomy for Cushing’s syndrome	1 (3.7)	^[32]^
Hyperemesis, vascular access	1 (3.7)	^[20]^
Hemodynamic deterioration	1 (3.7)	^[43]^
Congestive heart failure, pulmonary hypertension	1 (3.7)	^[14]^
Brain sarcoma	1 (3.7)	^[29]^

### Diagnosis

The timing of diagnosis of cardiac myxoma was described in 39 (76.5%) individuals. Two (5.1%) patients were diagnosed with a cardiac myxoma in the 1^st^ and 23^rd^ months before the current pregnancy, and their gestational ages were recorded as "-4" and "-92" weeks^[2,13]^. Among the 37 (94.9%) patients, the cardiac myxoma was diagnosed in the first, second and third trimesters in 7 (18.9%), 19 (51.4%) and 11 (29.7%) patients, respectively (*χ*^2^=9.1, *P*=0.011) with a mean of 21.7±8.4 (range, 6.7-38; median, 22) weeks (*n*=35).

The diagnostic techniques for the cardiac myxoma were described in 34 (66.7%) patients, by transthoracic echocardiography in 26 (70%)^[2,8-13,17,20,21,24,27-29,33-35,38,40,42,43,45,47,48,50]^, transthoracic and transesophageal echocardiography in 4 (13.3%)^[15,27,30,32]^, transesophageal echocardiography in 2 (6.7%)^[36,41]^, cardiac catheterization^[14]^ and a battery of tests^[46]^ in 1 (3.3%) patient.

Location of cardiac myxoma was not clearly stated in 5 (9.8%) patients. In the remaining 46 (90.2%) patients, it was located in the left atrium in 37 (80.4%) [intraatrial septum in 11 (29.7%)^[8-10,26,29,30,40,42,47,48]^, anterior mitral leaflet in 2 (5.4%)^[2,32]^, free wall^[36]^ in 1 (2.7%) and unknown in 23 (62.2%) patients^[2,13,14,16-18,23,24,26-28,31,33,34,37-39,44-46,50]^, right atrium in 5 (10.9%)^[2,11,15,20,36]^ (one was multiple^[20]^), left ventricle in 2 (4.3%)^[12,21]^, right ventricle in 1 (2.2%)^[43]^ and multiple sites (septal tricuspid leaflet, left atrium, intraatrial septum and left atrial appendage) in 1 (2.2%) patient^[41]^. Three patients had recurrent cardiac myxomas^[12,35,41]^ and one of them were recurrent multiple cardiac myxomas^[41]^. A total of 12 atrial myxomas including 11 (29.7%, 11/37) left atrial myxomas and 1 (20%, 1/5) right atrial myxoma^[20]^ prolapsed into the ventricle during diastole.

The attachment of the myxoma was described in 16 (31.4%) patients, it was pedunculated in 15 (93.8%)^[8,9,12-15,20,26,29,30,35,36,40,42,48]^ and sessile in 1 (6.3%) patient^[2]^. The dimensions of the cardiac myxomas were available in 25 (49.0%) patients. One of them was described as "egg-sized"^[46]^ and average dimension of the remaining 24 myxomas was 55.1±24.5 (range, 22-130; median, 55) mm (*n*=24)^[2,8,9,11,12-15,20,21,23,26,27,29,30,32,35,39,42,43,47]^.

The diagnosis of the cardiac myxoma was delayed in 3 (5.9%) patients in the 1^st^, 3^rd^ and 3^rd^ week after admission^[14,33,49]^.

### Treatment

Treatment was not given in 2 (3.9%) patients^[22]^. Concerning the remaining 49 patients, surgical operation was not performed in 2 (4.1%) patients but with anticoagulant and antibiotic therapy in one^[36]^ and decline of treatment by another^[41]^. A surgical resection of cardiac myxoma was performed in 47 (95.9%) patients including a sole cardiac myxoma resection in 41 (87.2%)^[2,8-21,23-27,29,31-39,43-46,48-50]^, concurrent mitral valve repair^[40,47]^ and staged mitral valve replacement^[28,29]^ in 2 (4.3%), and staged mitral valve repair^[30]^ and concurrent patent fossa ovalis closure^[42]^ in 1 (2.1%) patient. Cardiac surgery was performed in the first, second and third trimester in 5 (13.9%), 14 (38.9%) and 17 (47.2%) patients (*χ*^2^=9.8, *P*=0.008) at a mean of 25.2±9.4 (range, 9-41; median 27) weeks of gestational age (*n*=36); while timing of cardiac surgery was not given in 15 (29.4%) patients. A cardiac myxoma resection was delayed in 4 (7.8%) patients for a few^[43]^, 7^[21]^, 15^[40]^ and 690 days^[13]^, respectively. The feto-neonatal fate was not mentioned in 13 (25.5%) cases^[15,22,24,27,31,37,38,41,44,47]^. Voluntary termination of pregnancy was done in 7 (13.7%) cases with 6 (85.7%) early-mid-termed^[2,9,12,23,42]^ and 1 (14.3%) late pregnancy termination^[50]^ with mean gestational age of 17.0±8.3 (range, 11-31; median, 14) weeks (*n*=5). Delivery modes of remaining 31 (60.8%) pregnant patients were cesarean section in 19 (61.3%)^[2,10,11,13,16,20,21,28-30,32,35,36,39,40,43,45,46]^, vaginal delivery in 6 (19.4%)^[8,25,26,48,49]^, forceps under epidural analgesia in 1 (3.2%)^[33]^ and unknown in 5 (16.1%) patients^[14,17-19,34]^. One (3.2%) delivery timing was not given, 2 (6.5%) deliveries were in second trimester and 28 (90.3%) in third trimester with mean gestational age of 35.3±4.6 (range, 22-42; median, 37) weeks (*n*=30).

All the pregnant patients in the pregnancy termination group received a surgical resection of the cardiac myxoma with an isolated cardiac myxoma resection in 5 (71.4%), myxoma resection with patch repair of the iatrogenic septal defect in 1 (14.3%) and myxoma resection with patent fossa ovalis closure in 1 (14.3%) patient. One (14.3%) patient had pregnancy termination performed before cardiac surgery in 3 (42.9%), after the cardiac surgery in 2 (28.6%) and unknown surgical sequence in 2 (28.6%) patients. The indications for pregnancy termination was maternal pulmonary edema in 1 (14.3%)^[9]^, fetal growth retardation (fetal short femur) in 1 (14.3%)^[50]^ and unknown in 5 (71.4%) patients.

In the pregnancy termination group, 6 (85.7%) pregnant patients were event-free and 1 (14.3%) was complicated with postoperative transient acute myocardial infarction^[9]^. The prognosis of the pregnant patient was not given in 2 patients^[22]^. In the remaining 42 pregnant patients with a childbirth, 35 (83.3%) were event-free, 6 (14.3%) were complicated, with cerebral involvement (psychomotor agitation and mydriasis anisocoria)^[40]^, heart block^[30]^, pacing dependent rhythm^[28]^, pulmonary edema^[32]^, uterine contractions^[26]^, and premature labor^[34]^), and 1 (2.4%) was recurrent (requiring reoperation)^[29]^. No pregnant patients died. Pregnant patients' event free survival (*P*=0.680), complication (*P*=0.686) and recurrence rates (*P*=0.857) did not differ between pregnancy termination and delivery groups.

Eighteen (42.9%) patients had a delivery before cardiac surgery^[8,14,17,19,24,26-30,33,34,38,40,46,47,49]^, in the first, second, third and unknown trimester in 2 (11.1%), 8 (44.4%), 6 (33.3%) and 2 (11.1%) patients with a mean gestational age of 21.6±6.2 (range, 10-28.1; median, 23) weeks (*n*=16). Thirteen (31.0%) patients has cardiac myxoma resection performed after delivery in second, third and unknown trimester in 1 (7.7%), 9 (69.2%), and 3 (23.1%) patients at a mean gestational age of 34.2±5.5 (range, 22.2-41; median, 32.7) weeks (*n*=10)^[11,13,16,18,20,21,29,32,35,36,39,41,43]^. Two (4.8%) patients received a one-stage delivery and cardiac surgical procedure in the 31^st^ and 32.7^th^ week, respectively. Surgical sequence was unknown in 9 (21.4%) patients.

### Prognosis

In the delivery group, delivery mode was not given in 16 cases. Among the 26 deliveries with either cesarean section or vaginal delivery, 20 (76.9%) were event-free survivals, 4 (15.4%) were complicated and 2 (7.7%) died. Neonatal prognoses did not differ between delivery modes, treatment options, timing of cardiac surgery and sequence of cardiac myxoma in relation to delivery (Tables 4-7).

**Table 4:** Neonatal prognosis subjected to different delivery modes (Fisher exact test).

**Delivery mode**	**Total**	**Event-free**	**Complicated**	**Died**
Cesarean section, *n* (%)	19 (100)	15 (78.9) ^[2,10,16,20,21,28-30,35,36,39,40,45,46]^	2 (10.5) ^[13,29]^	2 (10.5) ^[11,32]^
Vaginal delivery, *n* (%)	6 (100)	4 (66.7) ^[5,25]^	2 (33.3) ^[5]^	0 (0)
Device delivery, *n* (%)	1 (100)	1 (100) ^[33]^	0 (0)	0 (0)
χ^2^	29.9	0.7	2.0	0.8
*P* value	0.000	0.705	0.366	0.671

**Table 7:** Neonatal prognosis according to sequences of cardiac myxoma resection and delivery (Fisher exact test).

**Sequence of cardiac myxoma resection**	**Total**	**Event-free**	**Complicated**	**Died**
Before delivery	18 (100)	15 (83.3) ^[8,14,17,19,24,26-28,30,34,38,40,46,47,49]^	3 (16.7) ^[26,29,33]^	0 (0)
After delivery	12 (100)	8 (66.7) ^[16,18,20,21,29,35,36,39,41]^	1 (8.3) ^[13]^	3 (25) ^[11,12,32]^
One-stage	2(100)	2 (100) ^[10,45]^	0 (0)	0 (0)
χ^2^	19.5	1.8	0.8	5.5
*P* value	0.000	0.413	0.683	0.063

Nomimal regression analysis showed that timing of delivery, delivery mode, surgical resection of the cardiac myxoma, simple or complex cardiac surgery, timing of cardiac surgery, sequence of cardiac surgery in relation to delivery and maternal complications were not predictive risk factors responsible for fetal outcomes.

## DISCUSSION

Cardiac myxoma is rare in pregnant patients. The diagnosis and management can be challenging in terms of the nature of the intracardiac mass, timing of delivery, necessity of cardiac surgery and risks of subsequent treatment^[36]^. The clinical manifestations of a cardiac myxoma can be one or more of the Goodwin's triad^[51]^. Patients may present with fatigue and dyspnea, which, however, can be misinterpreted as asthma or normal fatigue associated with pregnancy^[39]^. Echocardiography remains the standard non-invasive diagnostic modality, particularly in the pregnant patient^[52]^. In some patients, atrial thrombi may have a stalk and may be mistaken for myxomas, leading to unnecessary and potential harmful surgery^[53]^. A left intraatrial mass can be diagnosed as thrombus if associated with atrial fibrillation, dilated left atrium, mitral or tricuspid stenosis, low ejection fraction, prosthetic mitral or tricuspid valves, or spontaneous atrial contrast echoes^[54]^. Moreover, in the pregnant patients, cardiovascular magnetic resonance imaging is indicated for visualizing coarctation, aortitis, aortic dissection and atrial myxoma^[52]^.

Surgical management of cardiac myxoma is similar to that of the valvular disorders in the pregnant patients, even with minimally invasive cardiac surgical techniques^[55]^. Nevertheless, surgical indications of both conditions can be somehow different from each other. Congestive heart failure as a consequence of rheumatic mitral stenosis is always a contraindication of pregnancy. But it may be curable to percutaneous inteventional therapy, however, carrying the risk of fetal teratogenicity by manipulation under X-ray. Or else, an urgent valvular operation is warranted in the presence of infective endocarditis, intramural thrombus, paravalvular leakage, stuck prosthetic valve or thrombus formation. Meanwhile, the indications for cardiac myxoma resection are the potential embolic events and sudden death caused by myxoma-obstructed valve orifice^[2]^. Wang et al.^[2]^ reported three pregnant patients with a cardiac myxoma, two of which were complicated with cerebral infarctions and an urgent cardiac myxoma resection with later curettage was performed. Liu et al.^[6]^ described a pregnant patient with a cardiac myxoma presented with both cerebral infarction and central retinal artery occlusion, and a cardiac surgical resection was performed without delay. As for the potential of cardiogenic embolic events and possible preterm delivery due to hemodynamic changes, a timely surgical resection of cardiac myxoma can be indispensable during pregnancy.

The favorable maternal and fetal outcomes suggest that there might be a subset of pregnant patients with intracardiac masses who may benefit from non-surgical management^[36]^. Open heart surgery as well as the use of cardiopulmonary bypass may cause premature labor and endanger the baby^[49]^. The surgical resection of cardiac myxoma may be associated with a 30% baby loss rate, or postnatal physical or developmental disabilities^[39]^. It is encouraging that maternal survival rate was 100% in the pregnant patients with a cardiac myxoma, superior to that of the pregnant patients with infective endocarditis^[3]^. This might be interpreted as the results of the advantaged cardiopulmonary bypass techniques including high flow rate, high perfusion pressure and pulsatile flow applied in cardiac surgery during pregnancy^[30]^. By comparison, infective endocarditis and acute aortic dissection might be more dangerous to the pregnant patients than cardiac myxoma as for the infective nature of the former and the use of profound hypothermic circulatory arrest for the operation of the latter^[3,56]^. The present study also revealed that timing of delivery other than the delivery mode (by excluding early termination of pregnancy) and time sequence of cardiac surgery and delivery was closely related to feto-neonatal mortality. Cardiac surgery under cardiopulmonary bypass should be avoided in the first trimester, particularly after six weeks, due to the risk of teratogenesis^[50]^. The pregnant patients may wait for a few weeks^[13]^, or take weekly thyrotropin-releasing hormone and ß-methasone therapies for fetal lung maturation^[35]^. Precautions during cardiac operation include using blood priming solution, normothermic cardiopulmonary bypass and high perfusion pressure^[38]^.

Possible bias may be generated in present patient setting due to limited data available from the literature for the statistical analysis. Therefore, more abundant information of such patients is necessary for further precise results.

## CONCLUSIONS

Cardiac myxoma is rare in pregnant patients. In most cases, the cardiac myxoma is diagnosed in the second trimester and is resected in the third. Cesarean section was the most frequent delivery mode. The 100% maternal survival of this patient setting is encouraging. A delivery at early gestation was closely related to an increased feto-neonatal mortality. A delivery postponed to late pregnancy until fetal maturity may improve the feto-neonatal survival. In brief, embolic potential and hemodynamic deterioration are indications for an urgent cardiac myxoma resection. Otherwise, cardiac surgery should be avoided in the first trimester and be postponed until fetal pulmonary maturation or after delivery.

**Table:** 

**Authors’ roles & responsibilities**	
SMY	Study conception and design; analysis and/or interpretation of data; manuscript writing.

## Figures and Tables

**Table 5:** Neonatal prognosis of different treatment options (Fisher exact test).

**Treatment**	**Total**	**Event-free**	**Complicated**	**Died**
Surgical	39 (100)	31 (79.5)	5 (12.8)	3 (7.7)
Isolated myxoma resection	34 (100)	27 (79.4) ^[2,8,10,14,16-21,24,27,29,31,34,35,37-39,44-46,49]^	4 (11.8) ^[13,26,33,48]^	3 (8.8) ^[11,12,32]^
Myxoma resection with concurrent or staged additional cardiac surgery	5 (100)	4 (80) ^[28,30,40,47]^	1 (20) ^[29]^	0 (0)
Conservative	2 (100)	2 (100) ^[36,41]^	0 (0)	0 (0)
*P* value (surgical *vs.* conservative)	0.000	0.475	0.589	0.684
*P* value (myxoma resection *vs.* myxoma resection with concurrent or staged additional cardiac surgery)	0.000	0.976	0.607	0.489

**Table 6:** Neonatal prognosis according to timing of cardiac surgery (Fisher exact test).

**Timing of cardiac surgery**	**Total**	**Event-free**	**Complicated**	**Died**
1^st^ trimester	2 (100)	2 (100) ^[24,38]^	0 (0)	0 (0)
2^nd^ trimester	12 (100)	8 (66.7) ^[8,14,17,19,30,39,40,46]^	4 (33.3) ^[26,29,33,48]^	0 (0)
3^rd^ trimester	16 (100)	13 (81.3) ^[2,10,16,20,21,26,27,29,34,35,43,45,49]^	1 (6.3) ^[13]^	2 (12.5) ^[11,32]^
χ^2^	15.6	1.5	4.1	1.9
*P* value	0.000	0.480	0.132	0.392
